# PDGFR‐β and kidney fibrosis

**DOI:** 10.15252/emmm.201911729

**Published:** 2020-02-18

**Authors:** Alberto Ortiz

**Affiliations:** ^1^ IIS‐Fundacion Jimenez Diaz Department of Medicine School of Medicine Universidad Autonoma de Madrid Madrid Spain; ^2^ Fundacion Renal Iñigo Alvarez de Toledo‐IRSIN and REDINREN Madrid Spain

**Keywords:** Urogenital System

## Abstract

Chronic kidney disease (CKD) is one of the fastest growing global causes of death, estimated to rank among the top five by 2040 (Foreman *et al*, 2018). This illustrates current pitfalls in diagnosis and management of CKD. Advanced CKD requires renal function replacement by dialysis or transplantation. However, earlier CKD stages, even when renal function is still normal, are already associated with an increased risk of premature death (Perez‐Gomez *et al*, 2019). Thus, novel approaches to diagnose and treat CKD are needed. The histopathological hallmark of CKD is kidney fibrosis, which is closely associated with local inflammation and loss of kidney parenchymal cells. Thus, kidney fibrosis is an attractive process to develop tests allowing an earlier diagnosis of CKD and represents a potential therapeutic target to slow CKD progression or promote regression.

## Graphical abstract

A. Ortiz shares his views on the new model of primary renal fibrosis, based on transgenic mice with specific PDGFR‐β activation in renal mesenchymal cells, developed by P. Boor and collaborators (in this issue of *EMBO Molecular Medicine*).

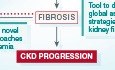

From the diagnostic point of view, tools for accurately staging CKD and for differentiating ongoing active kidney injury potentially responding to current therapies from therapy‐resistant residual fibrosis are suboptimal. Thus, although CKD risk categories are well defined and internationally accepted, they are based on estimated glomerular filtration rate (GFR) and assessment of albuminuria. GFR estimation is notoriously inaccurate, and there is no consensus on the risk associated with GFR‐based category G3a, the most common CKD stage (González‐Rinne *et al*, 2019). Additionally, pathological albuminuria is found both in active ongoing glomerular injury and in residual glomerular sclerosis. A kidney biopsy may help differentiate between active and inactive kidney diseases, but it is invasive and only provides information on a minuscule kidney sample, that may or may not represent the whole kidney. Thus, ongoing efforts aim at developing non‐invasive tools to assess global kidney fibrosis in the clinic. The most advanced approaches are urine proteomics and kidney magnetic resonance imaging (MRI), while molecular imaging of fibrosis‐specific proteins is still preclinical (Selby *et al*, 2018; Magalhães *et al*, 2017; Baues *et al*, 2019 online).

From the therapeutic point of view, there is an ongoing discussion on the active contribution of fibrosis to CKD progression, and thus, on whether targeting fibrosis may effectively slow or even reverse CKD; or on the contrary, if fibrosis represents a common end‐stage of any CKD, its targeting would not alter the natural history of CKD. Finally, fibrosis may even be a healing, beneficial process (Djudjaj & Boor, 2019). Clinical evidence on the active role of fibrosis allows several interpretations. On one hand, clinical trials exploring approaches directly targeting fibrosis have so far failed (Ramos *et al*, 2020). On the other hand, renin–angiotensin system (RAS) blockade, which constitutes the best established nephroprotective strategy, interferes with fibrosis along with other pathogenic processes.

In the present issue of *EMBO Molecular Medicine*, Buhl *et al* (2020) conclusively demonstrate that deregulated hyperactivity of the platelet‐derived growth factor receptor‐β (PDGFR‐β) in mouse renal mesenchymal cells leads to pathological proliferation of mesangial cells and interstitial fibroblasts. It further leads to a phenotype switch toward myofibroblasts driving mesangial sclerosis, interstitial fibrosis, decreased GFR, and renal anemia (Buhl *et al*, 2020). In short, PDGFR‐β overactivity in renal mesenchymal cells caused CKD. This preclinical model is clinically relevant since increased expression of PDGFR‐β by kidney mesenchymal cells is found in human CKD, and the features of this murine model overlap with those of human CKD. PDGFR‐β forms homodimeric or heterodimeric receptors for PDGF‐B and PDGF‐D, and targeting either PDGFR‐β, PDGF‐B, or PDGF‐D has been protective in diverse preclinical models of kidney disease. The originality of the present study is fourfold:

First, hyperactivity of a single receptor in mesenchymal cells only drove glomerular and interstitial fibrosis, and this preceded tubular atrophy and interstitial inflammation in the absence of hypertension, albuminuria or hematuria (Fig 1). Thus, the study demonstrates that fibrosis itself is pathogenic and may drive the full spectrum of CKD even in the absence of primary insult to parenchymal kidney cells or without engagement of common drivers of clinical CKD progression (proteinuria, hematuria, hypertension).

**Figure 1:**
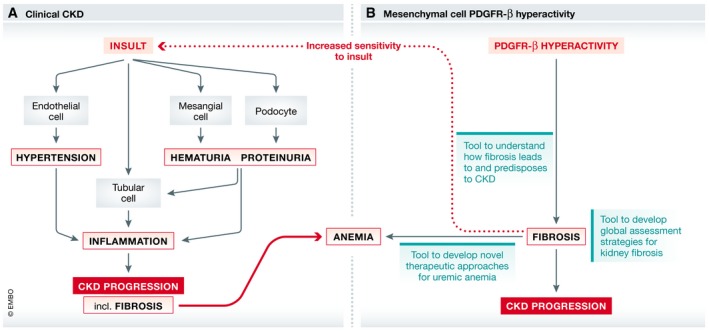
Clinical CKD progression versus kidney fibrosis and subsequent CKD progression induced by mesenchymal cell PDGFR‐β hyperactivity (A) A simplified view of clinical CKD progression is provided. Diverse insults may lead to primary injury of diverse kidney cell types, setting in motion clinical manifestation and processes (e.g., hypertension, hematuria, proteinuria) that amplify injury, usually by recruiting inflammatory mediators, leading to loss of parenchymal renal cells and kidney fibrosis. Uremic, EPO‐deficient anemia is a feature of advanced CKD. (B) Experimental kidney fibrosis induced by mesenchymal cell PDGFR‐β hyperactivity. Kidney fibrosis is the earliest manifestation of kidney injury, predisposes to CKD progression in response to other insults, and is associated with early anemia. Of note, fibrosis clearly precedes other features of CKD such as inflammation and parenchymal cell injury and is not associated with hypertension, hematuria, or proteinuria. The potential uses of this new tool are indicated.

Second, PDGFR‐β hyperactivity committed erythropoietin (EPO)‐producing interstitial cells to a fibrogenic phenotype at the expense of EPO production, thus driving anemia. This identifies PDGFR‐β as a negative regulator of physiological endocrine kidney EPO, which differs from the tumor microenvironment situation, where PDGF‐BB signaling via PDGFR‐β in local stromal cells induces EPO production. This may promote tumor growth through paracrine stimulation of tumor angiogenesis and by endocrine stimulation of extramedullary hematopoiesis (Xue *et al*, 2011). This finding opens the door to adjunctive therapies for uremic anemia that target PDGFR‐β hyperactivity in diseased kidneys. In this regard, it is significant that imatinib, an inhibitor of multiple receptor kinases, including PDGFR‐β, reversed anemia in mice with mesenchymal cell PDGFR‐β hyperactivation (Buhl *et al*, 2020). Indeed, anemia is a common adverse effect of imatinib used for cancer treatment.

Third, myofibroblast cell number and interstitial fibrosis (but not glomerular sclerosis) abnormalities were reversed by imatinib. However, whether there is an immediate clinical translation of this observation remains to be demonstrated. Indeed, the impact of imatinib on GFR was not addressed, and therapeutic use for human disease may be limited by the lack of impact on glomerular fibrosis, since glomerular health is a key determinant of GFR. Additionally, at the doses currently used in human cancer, imatinib has been associated with an increased incidence of acute kidney injury and chronic loss of GFR. Further research is thus warranted to define potentially nephroprotective imatinib regimens or to identify the specific additional kinases targeted by imatinib that may preclude nephroprotection in humans. While PDGFR‐β targeting with current tools may have limitations, Buhl *et al* identified the early signaling pathways engaged by PDGFR‐β overactivity in mesenchymal kidney cells. Interferon‐related signaling and JAK/STAT signaling were prominently represented. However, JAK/STAT signaling was not involved in kidney fibrosis in this model. This is important information since the JAK/STAT inhibitor baricitinib decreased albuminuria in diabetic kidney disease trials, although clinical development appears to have stalled.

Finally, a pure kidney fibrosis model may help set up kidney imaging or proteomics/metabolomics fingerprints to assess kidney fibrosis that are only modified by fibrosis, without being modified by concomitant kidney parenchymal cell injury or inflammation that represent confounding factors usually concurring with fibrosis in the clinic and in most available preclinical models. As an example, late gadolinium enhancement, long thought to represent irreversible scar tissue in cardiac MRI, is now also considered to be present during cardiac inflammation.
